# Autonomous chemo-metabolic construction of anisotropic cell-in-shell nanobiohybrids in enzyme-powered cell microrobots

**DOI:** 10.1126/sciadv.adu5451

**Published:** 2025-06-25

**Authors:** Nayoung Kim, Sang Yeong Han, Hyeong Bin Rheem, Hojae Lee, Insung S. Choi

**Affiliations:** ^1^ Department of Chemistry, KAIST, Daejeon 34141, Korea.; ^2^ Department of Chemistry, Hallym University, Chuncheon 24252, Korea.

## Abstract

Living organisms use intricate strategies to adapt and survive in response to potentially lethal environment changes. Inspired by cryptobiosis in nature, researchers have pioneered approaches to create cell-in-shell nanobiohybrids, aiming to endow cells with enhanced protection and exogenous functions. Yet, these methods still lack the biological autonomy intrinsic to natural cellular responses. Here, we present an innovative chemo-metabolically coupled strategy for the autonomous construction of cell-in-shell structures in cell growth medium. Our system harnesses ethanol fermentation by *Saccharomyces cerevisiae*, chemically coupled with an enzymatic cascade involving alcohol oxidase and horseradish peroxidase, to drive the nanoshell formation of polydopamine. The integration of autonomous shell formation with cellular proliferation produces anisotropic cell-in-shell structures, which can serve as enzyme-powered cell microrobots, upon conjugation with urease. Our autonomous system enables the creation of cell-in-shell nanobiohybrids with dynamic and adaptive environmental interactions, paving the way for transformative applications in synthetic biology, such as artificial cells, as well as advancements in cell-based therapies.

## Teaser

Integration of ethanol fermentation with chemical shell formation leads to autonomous creation of cell-in-shell nanobiohybrids.

## INTRODUCTION

Cell-in-shell nanobiohybrids have emerged as pivotal entities of interest across a spectrum of disciplines, encompassing cell therapy (*1*–*8*), renewable energy (*9*–*13*), cell factory engineering (*14*, *15*), microbiome research (*16*–*18*), and agricultural innovation (*19*). Cell-in-shell structures use artificial shells as protection checkpoints, shielding the nanoencapsulated cells from diverse physicochemical and biological stresses, including immunological attacks, elevated temperature, and ultraviolet (UV) irradiation (*20*–*23*). Concurrently, the nanoshells exhibit permselectivity, enabling the transport of essential small molecules such as nutrients and gases, thereby supporting the viability and functionality of the nanoencapsulated cells. Furthermore, the incorporation of functional entities, including enzymes, drugs, nanomaterials, and polymers, within nanoshells provides enhanced functionalities beyond the biochemical capabilities of wild-type cells (*15*, *24*–*32*). For example, the anchoring of exogenous enzymes onto Fe^3+^–tannic acid (TA) shells (*33*) nanoencapsulating bacteria (*Escherichia coli* and *Gluconobacter oxydans*) enables cooperative catalysis between enzymatic reactions and cellular metabolic activity for one-pot compound synthesis (*32*). In addition to the shell functionalization, functional entities can also be formed intracellularly, allowing nanobiohybrid structures to serve as multifunctional drug delivery systems. A salient example is the dual functionalization of *Saccharomyces cerevisiae* with calcium carbonate (CaCO_3_), wherein intracellular CaCO_3_ is generated by coupling with a metabolic byproduct (i.e., CO_2_) to act as a reservoir for therapeutic agents [e.g., doxorubicin (*34*) and curcumin (*35*)], while asymmetric, uniaxial CaCO_3_ crystallization on the cell surface enables self-propelled motion in gastric acid, offering the potential for deep penetration into the gastric mucus and enhanced drug accumulation in the stomach wall tissue (*35*, *36*).

While numerous methods have been devised in single-cell nanoencapsulation (SCNE) to construct cytoprotective and functional nanoshells around individual living cells, these approaches typically rely on the external chemical processes that operate entirely independently from cellular metabolism and activities. Consequently, they lack the biological autonomy observed in cellular responses to environmental changes. A notable example in nature is *Cryptococcus neoformans*, a fungal pathogen that produces a protective and functional melanin coat in response to nutrient deprivation (*37*, *38*). The melanin coat, synthesized through the enzymatic action of laccase using exogenous dihydroxyphenyl compounds such as l-3,4-dihydroxyphenylalanine, provides robust protection against oxidative damage from host effector cells, radiation, extreme temperatures, and other stressors, enhancing the organism’s survival and even virulence (*39*–*41*).

**Fig. 1.**
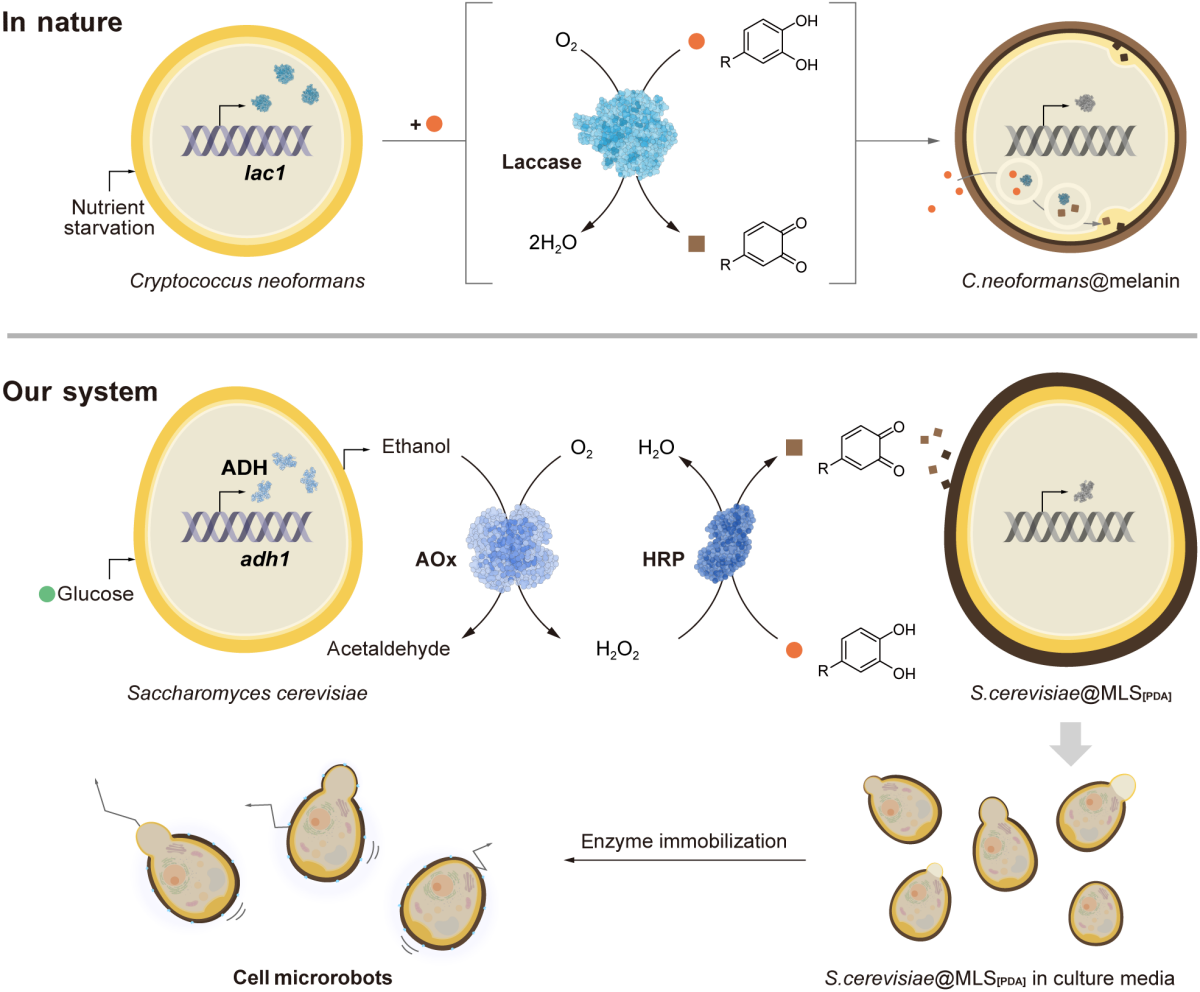
Schematic for autonomous chemo-metabolic construction of cell-in-shell nanobiohybrids as a mimic of biological autonomy in nature. In nature, *C. neoformans*, as a representative example, expresses laccase under nutrient-deprived conditions and forms melanin coats from external melanin precursors. In our system, MLS_[PDA]_ shells are autonomously formed on individual *S. cerevisiae*, where metabolic activity (i.e., alcohol fermentation) is coupled with shell-forming enzymatic actions of AOx and HRP. The metabolically coupled SCNE, conducted under cell-proliferating conditions, generates cell-in-shell structures with varying degrees of MLS_[PDA]_ coverage, functioning as enzyme-powered cell microrobots. *lac1*, laccase gene; *adh1*, alcohol dehydrogenase (ADH) gene.

Inspired by the self-sufficient defense mechanisms observed in *C. neoformans* and other cryptobiotic organisms in nature, this work develops autonomous chemical systems for nanoshell formation by harnessing cellular metabolic products. Specifically, we showcase the integration of alcohol fermentation in *S. cerevisiae* with an exogenous catalytic cascade reaction involving alcohol oxidase (AOx) and horseradish peroxidase (HRP), using 2-(3,4-dihydroxyphenyl)ethylamine (dopamine) as a precursor for shell formation in the autonomous SCNE process (Fig. 1). These intricately engineered shells are further tailored for the development of enzyme-powered cell microrobots, representing a notable advancement at the interface of biotechnology, nanoscience, and biomedical engineering (*42*–*48*).

## RESULTS

### Quantification of ethanol produced in *S. cerevisiae*

We first investigated ethanol (C_2_H_5_OH, EtOH) production during yeast fermentation using a two-step cascade reaction. The reaction involved AOx-catalyzed oxidation of EtOH to acetaldehyde (C_2_H_5_OH + O_2_ → CH_3_CHO + H_2_O_2_), followed by the HRP-mediated reduction of H_2_O_2_ to H_2_O in the presence of ABTS [2,2′-azino-bis(3-ethylbenzothiazoline-6-sulfonic acid)] as a probe (fig. S1A). Steady-state kinetic analysis yielded a plot correlating the initial reaction rate with EtOH concentration ([EtOH]), which conformed to the Michaelis-Menten model (fig. S1B). The maximum rate (*V*_max_) and Michaelis constant (*K*_m_) were determined to be 0.00682 absorbance s^−1^ (measured at 414 nm) and 31.8 mM, respectively. After establishing the reference curve, we incubated *S. cerevisiae* [OD_600_ (optical density at 600 nm) = 0.14] in a d-glucose solution ([d-glucose] = 55.5 mM) for a specified duration and subsequently analyzed the supernatant for the determination of [EtOH]. The amount of EtOH produced via fermentation showed a consistent linear increase over time: For instance, after 3 hours of incubation, [EtOH] was quantified at 3.02 mM (fig. S1C).

### Synthesis of melanin-like species via AOx-HRP system: Films and shells

Our initial investigation aimed to assess the effectiveness of the AOx-HRP system in the conversion of dopamine to melanin-like species (MLS_[PDA]_; PDA: polydopamine) within an EtOH-containing solution. The test was conducted with 3.02 mM EtOH—equivalent to the amount produced after 3 hours of yeast fermentation—and 3.0 mM dopamine after careful condition optimization for this work. The enzyme concentrations were set to be 1.0 U ml^−1^ for AOx and 0.2 U ml^−1^ for HRP, respectively. In our previous work at pH 8.5 (*49*), the MLS_[PDA]_-forming reaction was conducted with a much higher dopamine concentration (10 mM) for a longer reaction time (3 hours). Furthermore, the entire reaction process was repeated twice to ensure the construction of uniform shells. As a comparison, the present protocol employs milder reaction conditions, including a low dopamine concentration (3 mM), a more cytocompatible pH (7.4), and a shorter reaction duration (1.5 hours) in the autonomous SCNE (*5*, *6*).

We confirmed that the AOx-HRP system efficiently converted dopamine to MLS_[PDA]_ in the presence of EtOH in the solution, evident from the distinctive brown colorization observed in the reaction mixture and the corresponding UV-visible (UV-vis) absorption spectra (fig. S2A). The UV-vis spectra showed discernible peak increments at 480 and 410 nm, corresponding to aminochrome and dopamine-*o*-quinone species, respectively.

**Fig. 2.**
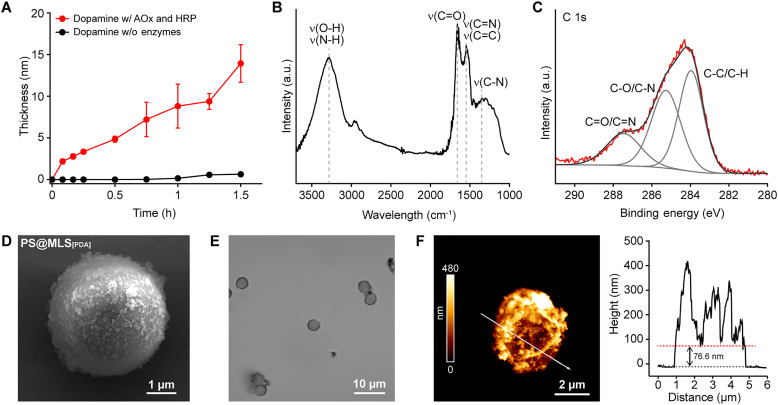
Characterizations of MLS_[PDA]_ films and shells. (**A**) Graph of MLS_[PDA]_ film thickness versus reaction time. The data are presented as mean values ± SD (*n* = 3, from independent experiments). (**B**) FTIR spectrum and (**C**) C 1s XPS spectrum of MLS_[PDA]_ film on Au substrates. (**D**) FE-SEM image of a PS microparticle after formation of the MLS_[PDA]_ shell. (**E**) DIC image of hollow MLS_[PDA]_ capsules after removal of sacrificial PS microparticles. (**F**) AFM image and height line profile of the hollow MLS_[PDA]_ capsules. The red dotted line indicates the height of bilayers. h, hours.

We also explored the material-independent formation of MLS_[PDA]_ films with flat gold (Au) substrates as a model, under identical reaction conditions ([EtOH] = 3.02 mM; [dopamine] = 3.0 mM; [AOx] = 1.0 U ml^−1^; [HRP] = 0.2 U ml^−1^). Ellipsometric analysis indicated that the enzyme system was essential for the film formation, producing a 14-nm-thick film after 1.5 hours of reaction (Fig. 2A). In contrast, minimal film formation (thickness < 1 nm) was observed in the absence of the enzymes under the same conditions. The successful formation of MLS_[PDA]_ films was confirmed through various characterizations, including Fourier transform infrared (FTIR) spectroscopy, x-ray photoelectron spectroscopy (XPS), field-emission scanning electron microscopy (FE-SEM), and contact angle measurements (Fig. 2, B and C, and fig. S2, B and C). For example, in the FTIR spectrum, the peaks corresponding to hydroxyl (O–H), carbonyl (C═O), and amine (C–N) groups in the PDA structure were observed at 3280, 1658, and 1346 cm^−1^, respectively (Fig. 2B). Additionally, the C 1s XPS peak was deconvoluted into three representative PDA peaks at 287.46 eV (C═O and C═N), 285.26 (C–O and C–N), and 283.96 (C–C and C–H) (Fig. 2C).

In addition, the formation of MLS_[PDA]_ shells was demonstrated with abiotic polystyrene (PS) microparticles (diameter: 3.97 μm). Briefly, particle suspensions were incubated for 1.5 hours in an aqueous solution of EtOH, dopamine, AOx, and HRP, with the concentrations of each component maintained as previously described. FE-SEM analysis showed that the particle surfaces became rougher, covered with MLS_[PDA]_ nanoparticulates (Fig. 2D and fig. S2D), thereby supporting the successful shell formation. Changes in ζ potential from −37.5 mV to −0.21 mV further confirmed the shell formation (fig. S2E). The durability of the MLS_[PDA]_ shells on PS particles allowed for the creation of hollow MLS_[PDA]_ capsules. When the core/shell PS particles were incubated in tetrahydrofuran (THF) for 24 hours, the PS cores were dissolved, resulting in hollow structures (Fig. 2E). Atomic force microscopy (AFM) analysis, performed through line profiling, indicated a shell thickness of approximately 40 nm (Fig. 2F). These results collectively confirmed that the amount of EtOH, produced from yeast fermentation of d-glucose, would be sufficient for the formation of MLS_[PDA]_ films and shells in the AOx-HRP system.

### Autonomous SCNE of *S. cerevisiae* via AOx-HRP system

We first applied the confirmed reaction protocol to form MLS_[PDA]_ shells on individual *S. cerevisiae*. In this setup, *S. cerevisiae* was preincubated for 3 hours in a d-glucose solution (OD_600_ = 0.28; [d-glucose] = 55.5 mM) before the shell formation process. After preincubation, a dopamine solution (final concentration: 3.0 mM) was added to the *S. cerevisiae* suspension, followed immediately by the introduction of the enzyme solutions (final concentration: [AOx] = 1.0 U ml^−1^; [HRP] = 0.2 U ml^−1^). It was anticipated that the metabolically released EtOH initiated the MLS_[PDA]_ shell-forming process. The overall reaction was carried out for 1.5 hours, after which the resulting *S.cerevisiae*@MLS_[PDA]_ was collected for characterization.

**Fig. 3.**
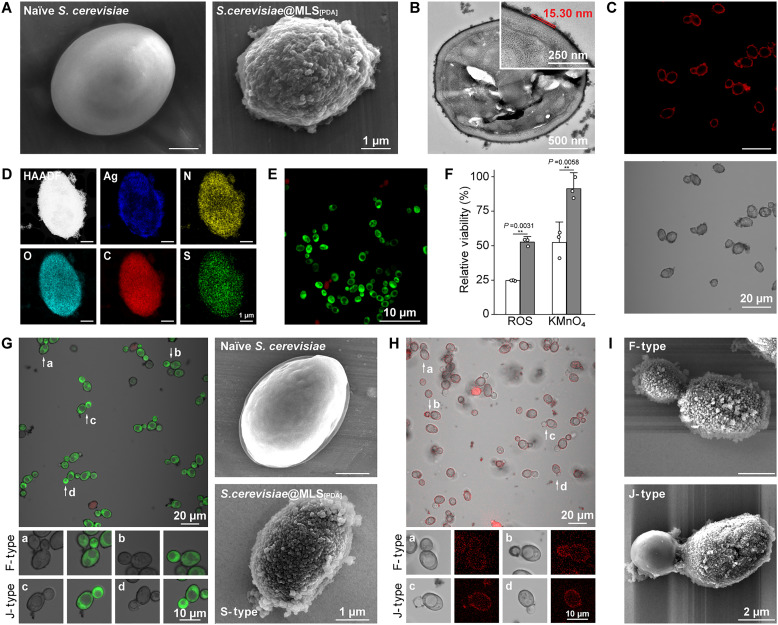
Autonomous chemo-metabolic SCNE of *S. cerevisiae*. (**A**) FE-SEM images of naïve *S. cerevisiae* and *S.cerevisiae*@MLS_[PDA]_ with preincubation of *S. cerevisiae* in a d-glucose solution. (**B**) TEM micrographs of ultramicrotome-sliced *S.cerevisiae*@MLS_[PDA]_. (**C**) CLSM images of BSA-647–labeled MLS_[PDA]_ shells, autonomously constructed without preincubation. Red: MLS_[PDA]_ shells labeled with BSA-647. (**D**) HAADF-STEM and EDS-TEM images of *S.cerevisiae*@MLS_[PDA]_ after incubation in a silver nitrate solution. (**E**) CLSM image of *S.cerevisiae*@MLS_[PDA]_ autonomously constructed without preincubation. Green, live; red, dead. (**F**) Viability of (white) naïve *S. cerevisiae* and (gray) *S.cerevisiae*@MLS_[PDA]_ after the exposure to ROS and KMnO_4_. The data are presented as mean values ± SD (*n* = 3, from independent experiments). Statistical analysis was performed using an unpaired Student’s *t* test (two-tailed) between two groups, giving *P* values, **P < 0.01. (**G**) Left: CLSM images of *S.cerevisiae*@MLS_[PDA]_ after autonomous SCNE in MM and (a to d) their enlarged images. Green, live; red, dead. Right: FE-SEM images of naïve *S. cerevisiae* and *S.cerevisiae*@MLS_[PDA]_ formed in MM. (**H**) CLSM images of BSA-647–labeled MLS_[PDA]_ shells and (a to d) their enlarged images with classifications into J-type and F-type. (**I**) FE-SEM images of F-type and J-type *S.cerevisiae*@MLS_[PDA]_, formed in MM.

FE-SEM and transmission electron microscopy (TEM) analyses showed the rougher cell surfaces composed of MLS_[PDA]_ nanoparticulates, confirming the successful shell formation under the conditions (Fig. 3, A and B). The observed difference in MLS_[PDA]_ shell thickness between PS microparticles and *S. cerevisiae* (40 nm versus 15.3 nm) is likely influenced by cellular activity and metabolic processes. During shell formation, *S. cerevisiae* actively consumes oxygen (O_2_) through respiration and fermentation (*50*), which may lead to localized O_2_ depletion in the reaction medium. Since AOx relies on O_2_ as a substrate for catalyzing dopamine oxidation, reduced O_2_ availability could affect its enzymatic efficiency, ultimately influencing the polymerization dynamics and the extent of shell deposition. In addition, doubling either the concentration of d-glucose or the concentrations of AOx and HRP did not result in noticeable variations in shell thickness, suggesting that MLS_[PDA]_ shell formation was chemo-metabolically coupled, with the alcohol fermentation of *S. cerevisiae* reaching saturation under the optimized protocol (fig. S3). The viability of *S.cerevisiae*@MLS_[PDA]_ was assessed with fluorescein diacetate (FDA) for viable cells (green) and propidium iodide (PI) for dead cells (red) as probes by confocal laser scanning microscopy (CLSM). The %viability was calculated to be 88.2% in comparison with naïve *S. cerevisiae* under identical reaction conditions (fig. S4A). The successful shell formation with metabolically released EtOH during preincubation suggested the feasibility of autonomous shell formation with EtOH generated in real time through alcohol fermentation.

We then undertook the task of autonomous construction of MLS_[PDA]_ shells without preincubation of *S. cerevisiae* with d-glucose, in which *S. cerevisiae*, d-glucose, dopamine, AOx, and HRP were mixed simultaneously at once. In a related study on autonomous shell formation in SCNE, MLS_[PDA]_ shells were generated around individual lactic acid bacteria, which released Mn^2+^ ions to catalyze the polymerization and deposition of dopamine in the medium (*51*). In the present setup, the cells grew and divided while fermenting d-glucose to produce EtOH, chemo-metabolically coupled with the MLS_[PDA]_ shell formation. The gradual color change of the reaction solution to dark brown indicated the utilization of EtOH released by *S. cerevisiae* for the MLS_[PDA]_ formation. FE-SEM analysis indicated the successful formation of MLS_[PDA]_ shell on individual *S. cerevisiae* (fig. S4B), and the TEM image of ultramicrotome-sliced *S.cerevisiae*@MLS_[PDA]_ also exhibited the MLS shells with an estimated thickness of approximately 20 nm (fig. S4C). Moreover, the visualization of MLS_[PDA]_ shells on cells was achieved through the binding of Alexa Fluor 647–conjugated bovine serum albumin (BSA-647; red) to the shells (Fig. 3C). In comparison, the cells incubated without the enzymes under the same reaction conditions did not show any noticeable red ring structures in the CLSM images (fig. S4D). The MLS_[PDA]_ shell formation was further supported by the MLS_[PDA]_-mediated reduction of Ag^+^ ions to silver nanoparticles at the cell surface (Fig. 3D). The Ag mapping signal appeared brighter at the outermost edge of *S.cerevisiae*@MLS_[PDA]_, whereas signals for N, O, C, and S did not exhibit this pattern.

The relative viability of *S.cerevisiae*@MLS_[PDA]_ was calculated to be 87.5%, confirming the cytocompatibility of our autonomous system (Fig. 3E). In comparison, the viability of *S. cerevisiae* SCNEd through PDA formation at pH 8.5 was reported to be about 54% (*49*), highlighting the high cytocompatibility of the metabolic coupling approach in the PDA-based SCNE. Furthermore, the cell division characteristics of *S.cerevisiae*@MLS_[PDA]_, monitored by measuring OD_600_ over time in yeast extract peptone dextrose (YPD) broth, showed minimal impact of the shell-forming process and MLS_[PDA]_ shells on cellular biofunctions when compared with naïve *S. cerevisiae* (fig. S5). In analogous to the protective role of melanin coats in *C. neoformans*, the MLS_[PDA]_ shells demonstrated protective effects on *S. cerevisiae* against oxidative stresses, such as reactive oxygen species (ROS) and potassium permanganate (KMnO_4_) (Fig. 3F). Specifically, after 0.5 hours of incubation in an aqueous solution of H_2_O_2_ (10 mM) and Fe^2+^ (5 mM), which generated ROS via the Fenton reaction, naïve cells exhibited a survival rate of only 24.7%. In stark contrast, more than half of *S.cerevisiae*@MLS_[PDA]_ (52.6%) survived under the same conditions. In addition, the viability of naïve *S. cerevisiae* dropped to 52.2% after 0.5 hours of incubation in a KMnO_4_ solution ([KMnO_4_] = 20 μM), whereas the viability of the SCNEd cells remained nearly unchanged (91.4%). No noticeable morphological changes of MLS_[PDA]_ shells were also observed after the treatments (fig. S6).

Living microorganisms play crucial roles in biomedical and biotechnological applications. However, their limited survival under harsh environmental conditions—such as high ionic strength, exposure to oxidizing agents, elevated temperatures, and adverse therapeutic settings—often leads to reduced viability and suboptimal functional outcomes. Our results showed that the MLS_[PDA]_ shell enhanced cellular robustness, supporting its potential for both in vitro and in vivo applications of cell-in-shell structures.

### Autonomous chemo-metabolic SCNE of *S. cerevisiae* in minimal medium

We further conducted autonomous MLS_[PDA]_ shell formation in minimal medium (MM), which contained only the essential nutritional components required for the growth of the wild-type strain, such as carbon sources and inorganic salts, serving as energy sources, cofactors in metabolic reactions, and buffering agents. Compared with the buffer solution with d-glucose, the MM better fulfills the nutritional requirements for cell proliferation. We first investigated the proliferation rate of *S. cerevisiae* under three different conditions: buffer solution without d-glucose [phosphate-buffered saline (PBS)], buffer solution with d-glucose (PBS + Glu), and MM (fig. S7). As expected, naïve cells proliferated for a longer period when incubated in MM, showing a continuous increase in cell density compared with those grown in PBS + Glu, where the increase in cell density diminished from 1.5 hours of incubation. Negligible cell growth was observed in the cells incubated in PBS.

For autonomous chemo-metabolic construction of MLS_[PDA]_ shells under cell-proliferating conditions, *S. cerevisiae* was mixed with a reaction solution containing MM, AOx, HRP, and dopamine, and the reaction was conducted for 1.5 hours. Similar to the reaction in PBS + Glu, the color of the mixed solution turned dark brown over time, indicating the MLS_[PDA]_ formation. The appearance of opaque cells with apparent edges in the CLSM images, along with rougher cell surfaces composed of nanoparticulates observed in the FE-SEM images, indicated successful construction of MLS_[PDA]_ shells on the individual cell surfaces (Fig. 3G). In addition, the cells subjected to the reaction in MM exhibited remarkably higher cell viability (98.4%) compared with the reaction in PBS (87.5%).

Consistent with the continuous increase in the cell density during incubation in MM, CLSM images showed budding *S. cerevisiae* cells with small daughter buds or daughter cells emerging from the mother cells. Closer examination revealed that the mother cell portions of the budding cells were coated with MLS_[PDA]_, while the smaller daughter cell portions exhibited varying degrees of MLS_[PDA]_ coverage. For textural simplicity, the cells, where both the mother and daughter portions were fully encapsulated, were classified as fully coated cells (F-type). In contrast, the cells, where the daughter portion appeared to have no coating, were classified as partially coated cells, or Janus cells (J-type). Fully coated single *S. cerevisiae* cells were classified as S-type. The CLSM images, taken after conjugation of BSA-647 to MLS_[PDA]_ shells, as well as FE-SEM images, further visualized and confirmed the formation of the shells with different coverages in the autonomous chemo-metabolic reaction (Fig. 3, H and I). The observed structural variations are likely due to inherent heterogeneity in cellular activity (*52*). In other words, the degree of MLS_[PDA]_ shell formation in MM is influenced not only by bulk reaction conditions but also by the local microenvironment experienced by individual cells. Specifically, variations in metabolic activity across the population—such as differences in cell proliferation rates and EtOH production during fermentation—would contribute to the coexistence of F-type and J-type cells. In addition, since daughter buds may experience different levels of exposure, as well as variations in the frequency and duration of exposure to MLS_[PDA]_ nanoparticulates, differences in shell formation are expected.

In a previous relevant study, J-structured *S. cerevisiae* cells were fabricated by precisely directing the uniaxial aggregation growth of CaCO_3_, formed from external Na_2_CO_3_ and CaCl_2_, on the cell surface (*35*, *36*). This process was regulated by the presence of cetyltrimethylammonium bromide and ethanol at the CaCO_3_ nucleation sites, and the cell viability was not reported. In another study, Janus modification of living *S. cerevisiae* was achieved through the deposition of TA-crosslinked Fe_3_O_4_ nanoparticles and the in situ growth of ZIF-67 metal-organic framework, after cell attachment to a poly(l-lysine)–modified surface (*53*). In addition, *S. cerevisiae* was asymmetrically nanoencapsulated within a non-crosslinked shell of mesoporous silica nanoparticles (MSNs) through a coupling reaction between aldehyde groups on MSNs and amino groups on the cell walls. Culturing the SCNEd cells in the YPD medium for 2 hours facilitated the generation of J-type cells through budding proliferation (*54*). While these seminal studies represent important advances in the asymmetric chemical modification of biological cells, our approach differs both conceptually and mechanistically, focusing on the integration of innate cellular activity with external chemical reactions to enable metabolically coupled shell formation.

Although reliable control over the proportion of different-symmetry cells remain challenging under the current experimental protocol, synergistic integration with other research domains may offer more effective strategies for regulating localized shell formation and symmetry distribution. For instance, microfluidic-based spatiotemporal regulation of cell exposure to MLS_[PDA]_ precursors and the external enzymes could enable precise modulation of shell formation dynamics (*55*–*57*), while the use of *S. cerevisiae* strains overexpressing alcohol dehydrogenase (ADH) or alternative cell lines with distinct metabolic profiles may further refine structural outcomes. Moreover, the post-separation of J-type cells after autonomous shell formation in MM could also be envisioned as a strategy for production of purified J-type cells (*58*, *59*).

### Construction of enzyme-powered cell microrobots

**Fig. 4.**
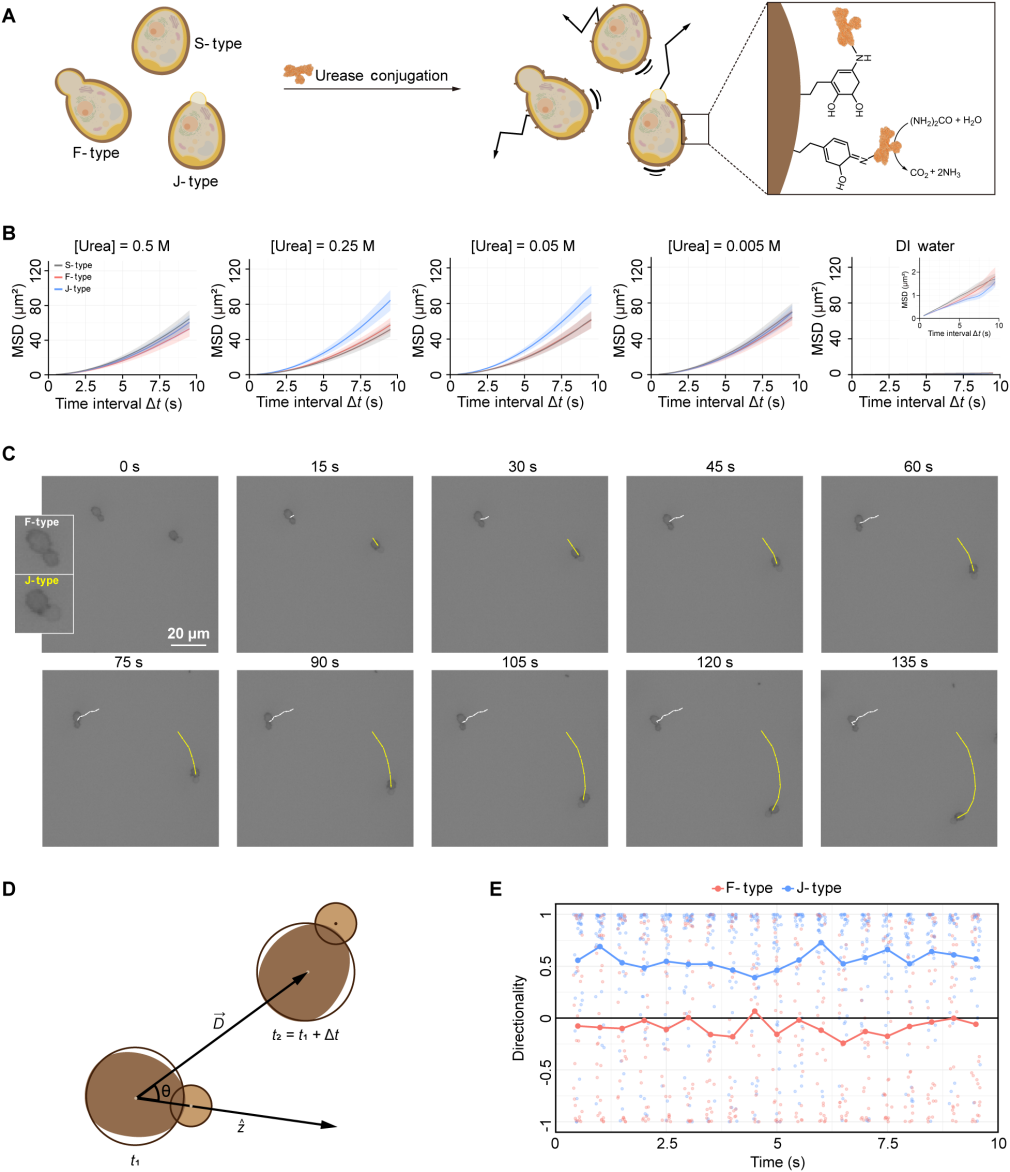
Construction of urease-powered cell microrobots and their motional behaviors. (**A**) Schematic for the formation of urease-powered cell microrobot from *S.cerevisiae*@MLS_[PDA]_ with conjugation of urease to MLS_[PDA]_ shells. (**B**) Graphs of MSD versus time interval for S-type, F-type, and J-type microrobots in urea solutions with different concentrations and DI water. The data are presented as mean values ± SE. In the case of 0.5 M, the number of particles (*n*) are 97, 76, and 69 for S-, F-, and J-types, respectively; for 0.25 M, 96, 84, and 87; for 0.05 M, 87, 64, and 84; for 0.005 M, 85, 90, and 80; in the case of DI water, 60, 74, and 39. (**C**) Time-lapse DIC images of self-propulsive motions of F-type and J-type microrobots in 0.05 M urea solutions. (**D**) Definition of directionality factor, cosine of the angle between the rod axis ( $\hat{z}$ ) and the direction of the microrobot movement ( $\vec{D}$ ). (**E**) Motion directionality of F-type and J-type microrobots in 0.05 M urea solutions.

Urease-powered cell microrobots were created by incubating the cells, SCNEd for 1.5 hours in MM, in a urease solution (4 mg ml^−1^, pH 7.4) for 18 hours, coupling them with urease via a Schiff-base reaction (Fig. 4A). According to the Bradford assay, approximately 73% of urease in the urease solution was conjugated to the MLS_[PDA]_ shells of the encapsulated cells. Beyond the Schiff-base reaction–mediated strategy, various chemical strategies have been used to functionalize abiotic nano/microrobots with enzymes while maintaining their activity and accommodating a range of surface properties (*60*–*62*). In addition to post-shell functionalization with enzymes, the in situ incorporation of enzymes directly into MLS_[PDA]_ shells during autonomous shell formation could also be envisioned.

In general, the enzymes immobilized on nano- and microobjects consume substrates and generate a local concentration gradient or electric field, which acts as a driving force for their self-propulsion. Urease-powered movement is fueled by the decomposition of urea into ammonia and carbon dioxide, which are further converted into diverse ionic species, such as NH_4_^+^, HCO_3_^−^, CO_3_^2−^, and OH^−^, in aqueous solution (*63*, *64*). The concentration gradient of these ionic species, along with the resulting local electric field, is suggested to propel the objects through a mechanism known as ionic self-diffusiophoresis (*65*). Motional behaviors of the cell microrobots were recorded using CLSM with a frame rate of 2 frames per second (fps). The initial cell density was adjusted approximately to 4 × 10^5^ cells ml^−1^. The solutions of cell microrobots were mixed with aqueous urea solutions of varying concentrations (final concentrations: 0.5, 0.25, 0.05, and 0.005 M). Specifically, three types of cells—S-type, F-type, and J-type—were analyzed to investigate the correlation between their coating symmetry (degree) and self-propulsion, where the budding *S. cerevisiae* consisted of two portions: a daughter cell portion connected to a mother cell portion. J-type and F-type cell microrobots were distinguished by differences in brightness between the mother cell portion and the daughter cell portion in the CLSM images with differential interference contrast (DIC) mode. To observe the brightness difference and accurately track the motional behaviors of the cells, it was necessary to ensure that the microrobots were clearly focused and not influenced by convective flows. Therefore, their motional behaviors were tracked 150 s after mixing, allowing the mixture of cells and urea to stabilize.

For each concentration of urea, the motional behaviors of each type of cell microrobots were tracked and analyzed. Using the *X*-*Y* coordinates of the trajectories obtained from optical tracking, the mean-squared displacement (MSD) was calculated as a function of the time interval ( ∆*t*). While the microrobots exhibited characteristic Brownian motion in deionized (DI) water, they showed enhanced directional propulsive motion in the aqueous urea solution. Quantitative analysis of their motional behaviors using particle tracking revealed that, for all types of microrobots, MSD values increased in the urea solution compared with DI water, and the shape of the MSD curves was quadratic (Fig. 4B). This quadratic curve suggested that the microrobots were actively moving in a directed manner rather than passively diffusing. The MSD values for F- and S-type cells were comparable across all tested urea concentrations. In contrast, J-type cell microrobots exhibited notably higher MSD values in the 0.25 and 0.05 M urea solutions, indicating their most effective directional movement. The directional motion observed for F- and S-type cells was attributed to local asymmetries in urease distribution, inducing directional propulsion rather than Brownian motion, consistent with the reported behavior of urease-functionalized symmetric polystyrene@SiO_2_ microrobots (*60*). Furthermore, naïve *S. cerevisiae* exhibited minimal motility, characterized by Brownian motion, in both DI water and urea solutions of varying concentrations, indicating that their intrinsic self-motion has a negligible impact on the motility and directionality of cell microrobots (fig. S8). The motional behavior of the cell microrobots was also examined in other media—including PBS, Dulbecco’s modified Eagle’s medium (DMEM), simulated gastric fluid, and simulated urine—showing reduced motility and lower MSD values in all cases, likely due to ionic species interfering with the formation of local electric fields necessary for propulsion, as suggested in a previous study (fig. S9) (*66*).

In the case of J-type cell microrobots, the uncoated parts faced the front of the movement (Fig. 4C). The correlation between the movement direction and the constructed shell was analyzed with the use of a directionality factor, defined as the cosine of the angle between the rod axis ( $\hat{z}$ ) and the direction of microrobot movement ( $\vec{D}$ ) (Fig. 4D) (*67*). The rod axis was calculated based on the center coordinates of the mother cell and the daughter cell of J-type and F-type microrobots, while the direction of microrobot movement was determined from center coordinates of the mother cell at each time interval. When a cell microrobot moved in the direction aligned with the axis from the mother cell to the daughter cell, it had a directionality of 1 ( cos0 ). As a comparison, the directionalities of J-type and F-type microrobots were calculated based on their trajectories over 9.5 s, with an analysis time interval of 0.5 s, in the 0.05 M urea solution. The analysis clearly confirmed the directed movement of J-type microrobots, which had a directionality factor of 0.55, while F-type microrobots had a directionality factor close to 0 (Fig. 4E). The results implied that the asymmetric propulsive force generated by uneven distribution of ureases, from asymmetrically formed MLS_[PDA]_ shells and the anisotropic shape of the cell microrobots, enhanced and strengthened their directional self-propulsive motions over the Brownian diffusive motions.

## DISCUSSION

It has been a long-standing goal to achieve autonomous systems where cell proliferation harmonizes with shell formation—mimicking the biological autonomy observed in cellular responses to environmental changes—in the development of functional cell-in-shell nanobiohybrids. To our knowledge, we present the first demonstration of such an autonomous construction of cell-in-shell structures by coupling cellular metabolic reactions with shell-forming process, facilitated by an exogenous catalytic cascade reaction. Ethanol, produced during the alcohol fermentation of *S. cerevisiae*, was used by the AOx-HRP cascade system to accelerate dopamine-based shell formation, resulting in highly functionalizable MLS_[PDA]_ shells. The autonomous approach, combined with the inherent heterogeneity of cellular activity, enabled the formation of anisotropic cell-in-shell structures, which were further adapted into enzyme-powered microrobots capable of self-propulsion. With the biocompatibility of MLS_[PDA]_ shells and their capacity to load various functional molecules, the metabolically coupled method developed in this study hold great promise for the fabrication of multifunctional cell-based structures including microrobots, which have potential applications in various sectors, including cell-based drug delivery, targeted cell therapy, and localized biocatalysis, bridging the fields of biotechnology, nanoscience, and biomedical research.

## MATERIALS AND METHODS

### Materials

Ethanol ( ≥99.9%, Sigma-Aldrich), urea ( ≥98%, Sigma-Aldrich), PBS (10 mM, pH 7.4, Welgene), potassium phosphate monobasic anhydrous (KH_2_PO_4_, ≥99.0%, Sigma-Aldrich), glycine ( ≥99%, Sigma-Aldrich), magnesium sulfate (MgSO_4_, 98.0%, Samchun), thiamine hydrochloride ( ≥99%, Sigma-Aldrich), AOx (from *Pichia pastoris*, Sigma-Aldrich), HRP (from *Armoracia rusticana*, Sigma-Aldrich), urease [from *Canavalia ensiformis* (Jack bean), Sigma-Aldrich], dopamine hydrochloride (Sigma-Aldrich), d-(+)-glucose ( ≥99.5%, Sigma-Aldrich), 2,2′-azino-bis(3-ethylbenzothioazoline-6-sulfonic acid) diammonium salt (ABTS; ≥99.5%, Sigma-Aldrich), PS microparticles (diameter: 3.97 μm, microParticles GmbH), THF (≥99.9%, Sigma-Aldrich), BSA–Alexa Fluor 647 (Thermo Fisher Scientific), FDA (Sigma-Aldrich), PI (Sigma-Aldrich), YPD broth (Duchefa Biochemistry), YPD agar (Duchefa Biochemistry), glutaraldehyde solution (25%, Sigma-Aldrich), lacey/carbon copper grids (200 mesh, Electron Microscopy Sciences), osmium tetroxide (Sigma-Aldrich), potassium hexacyanoferrate (II) trihydrate [K_4_[Fe(CN)_6_]·3H_2_O, Sigma-Aldrich], sodium cacodylate trihydrate [(CH_3_)_2_AsO_2_Na·3H_2_O, Sigma-Aldrich], propylene oxide (Sigma-Aldrich), lead (II) citrate tribasic trihydrate [(C_6_H_5_O_7_)Pb_3_·3H_2_O, Sigma-Aldrich], DMEM [with d-(+)-glucose (4500 mg liter^−1^), l-glutamine, sodium pyruvate (100 mg ml^−1^), and sodium bicarbonate, Welgene], fetal bovine serum (FBS; Welgene), penicillin-streptomycin [P/S; penicillin (5000 U ml^−1^) and streptomycin (5000 μg ml^−1^), Welgene], yeast from *S. cerevisiae* (Sigma-Aldrich), sodium chloride (NaCl; Daejung), potassium chloride (KCl; Samchun), calcium chloride dihydrate (CaCl_2_·H_2_O; Sigma-Aldrich), sodium sulfate (Na_2_SO_4_; Junsei), and ammonium chloride (NH_4_Cl; Sigma-Aldrich) were used as received. Au substrates were prepared by thermal deposition of Ti (5 nm) and Au (100 nm) onto silicon wafers (Sehyoung Wafertech). DI water (18.4 megohm·cm) from Milli-Q Direct 8 (Millipore) was used.

### Quantification of EtOH produced during yeast fermentation

#### Establishment of a reference plot

To quantify EtOH produced during yeast fermentation, a reference plot was formulated, correlating the initial reaction rate of an enzymatic cascade reaction involving AOx and HRP with EtOH concentration. EtOH solutions of varying concentrations (160, 80, 50, 40, 30, 20, 10, 5, 2.5, 1.25, 0.625, 0.3125, 0.15625, and 0.078125 mM) were prepared in PBS (pH 7.4). To 380 μl of PBS were added sequentially 100 μl of ABTS solution (20 mM), 10 μl of AOx (1 U ml^−1^), 10 μl of HRP (250 U ml^−1^), and 500 μl of each EtOH solution (final concentrations: [EtOH] = 80, 40, 25, 20, 15, 10, 5, 2.5, 1.25, 0.625, 0.3125, 0.15625, 0.078125, and 0.0390625 mM; [AOx] = 0.01 U ml^−1^; [HRP] = 2.5 U ml^−1^; [ABTS] = 2 mM). Absorbance of the reaction solution was measured at 414 nm every minute using a microplate reader (SpectraMax iD5, Molecular Devices). Initial reaction rates were calculated from the initial slopes of the time-dependent absorbance curves. The achieved reference curve was fitted to the Michaelis-Menten model, and maximum rate (*V*_max_) and Michaelis constant (*K*_m_) were determined.

#### Quantification of EtOH released by S. cerevisiae

A single colony of *S. cerevisiae* was picked from a YPD agar plate, suspended in YPD broth, and cultured in a shaking incubator at 33°C for 30 hours. The *S. cerevisiae* cells were washed with DI water three times and incubated in a d-glucose solution ([d-glucose] = 55.5 mM; OD_600_ = 0.14). After predetermined incubation times (0.25, 0.5, 0.75, 1, 1.5, 2, 2.5, 3, 4, 5, and 6 hours) in the shaking incubator, the solutions were centrifuged at 15,000*g* for 5 min, and supernatants were collected and diluted with PBS. To 380 μl of PBS were added sequentially 500 μl of the diluted supernatant, 100 μl of ABTS solution (20 mM), 10 μl of AOx (1 U ml^−1^), and 10 μl of HRP (250 U ml^−1^) (final concentrations: [AOx] = 0.01 U ml^−1^; [HRP] = 2.5 U ml^−1^; [ABTS] = 2 mM). Absorbance of the reaction solution was measured at 414 nm every minute using a microplate reader. Initial reaction rates were calculated, and EtOH concentrations in the supernatants were calculated based on the established reference plot.

### MLS_[PDA]_ film and shell formation via AOx-HRP system

Flat Au substrates were selected as a model for the construction of MLS_[PDA]_ films. Au substrates were immersed in a reaction solution ([EtOH] = 3.02 mM; [dopamine] = 3.0 mM; [AOx] = 1.0 U ml^−1^; [HRP] = 0.2 U ml^−1^) and stirred with 120 rpm at 33°C. PBS (pH 7.4) was used as the solvent. After predetermined reaction times (0, 5, 10, 15, 30, 45, 60, 75, and 90 min), the Au substrates were washed with DI water and dried under argon gas. Film thickness was measured with a spectroscopic ellipsometer (Elli-SE, Ellipso Technology Co.). To form MLS_[PDA]_ shells on PS microparticles, a 50-μl aliquot of the particle suspension in water (10% w/v) was centrifuged to obtain a pellet. To the pellet was added 16 ml of a reaction solution ([EtOH] = 3.02 mM; [dopamine] = 3.0 mM; [AOx] = 1.0 U ml^−1^; [HRP] = 0.2 U ml^−1^), and the reaction mixture was gently shaken for 1.5 hours at 33°C. After incubation, the solution was centrifuged at 500*g* to collect PS@MLS_[PDA]_ particles, which were then washed with DI water at least three times to remove any residual chemicals. Hollow MLS_[PDA]_ capsules were obtained by dissolving the sacrificial PS microparticle cores with THF. PS@MLS_[PDA]_ in THF was vortexed for 1 min and centrifuged at 2000*g*, followed by supernatant removal. This process was repeated, followed by a 24-hour incubation in THF to ensure complete dissolution of the PS microparticles. For characterization by FE-SEM and AFM, 5 μl of the hollow MLS_[PDA]_ capsule solution was air-dried on Au substrates and slide glasses, respectively.

### Autonomous chemo-metabolic SCNE of *S. cerevisiae*

A single colony of *S. cerevisiae* was picked from a YPD agar plate and cultured in YPD broth with shaking at 33°C for 30 hours. Before SCNE, the cells were washed with DI water three times. For SCNE with preincubation, *S. cerevisiae* was preincubated in 8 ml of d-glucose solution (OD_600_ = 0.28; [d-glucose] = 55.5 mM) for 3 hours at 33°C. After preincubation, 8 ml of reaction solution ([dopamine] = 6.0 mM; [AOx] = 2.0 U ml^−1^; [HRP] = 0.4 U ml^−1^) was added to initiate MLS_[PDA]_ formation (final concentrations: [dopamine] = 3.0 mM; [AOx] = 1.0 U ml^−1^; [HRP] = 0.2 U ml^−1^), and the reaction was carried out for 1.5 hours at 33°C. For SCNE without preincubation, to a pellet of *S. cerevisiae* was added directly the reaction solution containing d-glucose (final concentrations: [dopamine] = 3.0 mM; [AOx] = 1.0 U ml^−1^; [HRP] = 0.2 U ml^−1^; [d-glucose] = 55.5 mM), and the reaction was carried out for 1.5 hours at 33°C. To investigate the tunability of MLS_[PDA]_ shell thickness, reactions were carried out using either a doubled concentration of d-glucose ([d-glucose] = 111 mM) or doubled concentrations of both enzymes (final concentrations: [AOx] = 2.0 U ml^−1^; [HRP] = 0.4 U ml^−1^). For cell division test, the 1-ml suspension of naïve *S. cerevisiae* and *S.cerevisiae*@MLS_[PDA]_ (OD_600_: 0.15 each) was added to 150 ml of YPD broth medium (final OD_600_: 0.001) and cultured in a shaking incubator at 33°C. The cell growth culture medium (1 ml) was picked at the predetermined time for the OD_600_ measurement. For SCNE in MM, MM composed of d-glucose (55.5 mM), MgSO_4_ (10 mM), KH_2_PO_4_ (29.4 mM), glycine (13 mM), and thiamine-HCl (3 μM) was prepared using DI water with the final pH adjusted to 7.4 with 1 M NaOH solution (*68*). To a pellet of *S. cerevisiae* was added the reaction solution prepared with MM as the solvent (final concentrations: [dopamine] = 3.0 mM; [AOx] = 1.0 U ml^−1^; [HRP] = 0.2 U ml^−1^). The reaction solution was incubated for 1.5 hours at 33°C with gentle shaking. *S.cerevisiae*@MLS_[PDA]_ was collected by centrifugation and washed with DI water, and cell viability was assessed with FDA and PI. A 5-μl aliquot of FDA stock solution (10 mg ml^−1^ in acetone) and 2 μl of PI stock solution (1 mg ml^−1^ in DI water) were mixed with 1 ml of a cell suspension and incubated for 15 min at 33°C with shaking. To visualize the MLS_[PDA]_ shells, a cell pellet was suspended in 1 ml of Alexa Fluor 647–conjugated BSA solution (5 mg ml^−1^) in PBS (pH 7.4) for 18 hours at 33°C. After incubation, the cells were collected by centrifugation, washed with PBS, and analyzed by CLSM. For high-angle annular dark-field scanning transmission electron microscopy (HAADF-STEM), TEM, and FE-SEM imaging, cells were fixed in an aqueous solution of glutaraldehyde (2%) for 30 min and washed with DI water three times. After sequential dehydration with aqueous EtOH solutions [25, 50, 75, 90, 95, and three 100% (v/v) steps; 5 min each], a 5-μl aliquot of diluted cell suspension was air-dried on lacey/carbon copper grids for HAADF-STEM and TEM and Au substrates for FE-SEM overnight. For TEM analysis of ultramicrotome-sliced *S.cerevisiae*@MLS_[PDA]_, cells were sequentially fixed in 3% glutaraldehyde and 2% osmium tetroxide with 3% potassium hexacyanoferrate(II) trihydrate in 0.1 M cacodylate buffer at 4°C overnight. After several washes with DI water, pellets were dehydrated using a graded EtOH series (30, 50, 70, 80, 90, and 100% v/v), followed by propylene oxide for 10 min each, and then embedded in Epon 812 resin. After curing at 70°C for 36 hours, thin sections were prepared with an ULTRACUT UC7 ultramicrotome (Leica Biosystems, Vienna, Austria), mounted on 75-mesh copper grids, and counterstained with uranyl acetate and lead citrate for 10 min and 7 min, respectively. TEM images were obtained at 120 kV with a KBSI Bio-TEM (JEM1400 Plus). For cytoprotection studies against ROS and KMnO_4_, cell pellets were incubated in aqueous solutions of H_2_O_2_ (10 mM) and Fe^2+^ (5 mM) or KMnO_4_ solution (20 μM) for 30 min. After treatment, cells were washed with DI water three times, subjected to the FDA/PI staining, and analyzed by CLSM. Surface morphologies of MLS_[PDA]_ shells after the treatments were investigated via FE-SEM imaging.

### Construction of enzyme-powered cell microrobots

A pellet of *S.cerevisiae*@MLS_[PDA]_ was suspended in urease solution (4 mg ml^−1^ in PBS, pH 7.4) and incubated at 33°C for 18 hours with gentle shaking to immobilize urease on the MLS_[PDA]_ shells. Following incubation, the solution was centrifuged, and the collected cells were washed three times with DI water. Supernatant solutions were collected to determine the urease concentration based on the Bradford assay. A standard absorbance curve was prepared using urease solutions of varying concentrations (4, 2, 1, 0.5, 0.25, and 0.1 mg ml^−1^). For each measurement, 250 μl of Bradford reagent was mixed with 50 μl of urease solution, and the mixture was incubated for 30 min at room temperature. Absorbance at 595 nm was then measured with a microplate reader. The urease concentration in the supernatant solution was determined by measuring the absorbance and fitting the value to the standard curve. To calculate the percentage of urease conjugated to the MLS_[PDA]_ shells, the amount of urease in the supernatant was subtracted from the initial urease amount in the solution, providing an indirect determination of the conjugation efficiency.

### Motion analysis of cell microrobots

A CLSM 700 microscope with a 20× objective lens was used to observe and record the motional behaviors of cell microrobots. The density of the cell microrobots was adjusted approximately to 4 × 10^5^ cells ml^−1^ using a hemocytometer, with DI water as the solvent. A 260-μl aliquot of the microrobot solution was mixed with 260 μl of urea solution at varying concentrations (1, 0.5, 0.1, and 0.01 M) or with 260 μl of DI water. After thorough mixing, the solution was loaded onto a homemade chamber (fig. S10). Videos, typically 240 s in length, were captured with a photomultiplier tube (PMT) at a frame rate of 2 fps. Motion trajectories and corresponding coordinates of cell microrobots between 150 and 160 s post-mixing were analyzed using the TrackMate plugin in the ImageJ software. MSD was calculated from the obtained coordinates using the following equation: $\text{MSD}\left(∆t\right)=\langle {\left[x_{i}\left(t+∆t\right)-x_{i}\left(t\right)\right]}^{2}\rangle$ ( i=1,2 for two-dimensional analysis). The procedures were repeated for analyzing intrinsic motional behaviors of naïve *S. cerevisiae* in urea solutions at varying concentrations. Motional behaviors of cell microrobots were examined by using PBS, DMEM supplemented with 10% FBS and 1% PS, or simulated gastric fluid [0.2% (w/v) NaCl, pH 3] as a solvent for preparing 0.05 M urea solution, and by using simulated urine containing 0.05 M urea. The simulated urine, composed of NaCl (50 mM), KCl (21.5 mM), CaCl_2_ (7.62 mM), Na_2_SO_4_ (15.8 mM), KH_2_PO_4_ (10.3 mM), NH_4_Cl (18.7 mM), and urea (50 mM), was prepared according to the protocol described in previous reports (*42*, *69*). For directionality analysis, the center coordinates of the mother and daughter cell portions of the microrobots were tracked using the Manual Tracking plugin in the ImageJ software. On the basis of the mother and daughter cell coordinates at time t , the rod axis ( $\hat{z}$ ) was established. The direction of the microrobot movement ( $\vec{D}$ ) was determined from the mother cell coordinates at times $t_{1}$ and $t_{2}$ (where $t_{2}=t_{1}+∆t$ and ∆t=0.5s ), representing the displacement of the microrobot over one time interval. The directionality factor was defined as the cosine of the angle between the rod axis ( $\hat{z}$ ) and the movement direction ( $\vec{D}$ ), and calculated for every time point over 9.5-s duration.

### Characterizations

FE-SEM imaging was performed with an FEI Inspect F50 microscope with accelerating voltage of 10 kV after sputter-coating with platinum. Film thickness was measured with a spectroscopic ellipsometer Elli-SE (Ellipso Technology). Measurement of UV-vis absorbance and analysis of enzyme kinetics were performed with a microplate reader (SpectraMax iD5, Molecular Devices). FTIR spectra were obtained with a Nicolet Nexus FTIR spectrophotometer (Thermo Fisher Scientific). HAADF-STEM imaging and corresponding energy-dispersive x-ray spectroscopy (EDS) elemental mapping were performed with a Talos F200X (FEI) operated at 200 kV. AFM images were obtained with a NanoWizard 4 BioAFM (JPK). ζ potentials were measured with a Zetasizer Nano ZS (Malvern). TEM images of ultramicrotome-sliced cells were obtained using a KBSI Bio-TEM (JEM1400 Plus at 120 kV; JEOL Ltd., Tokyo, Japan).

### Statistical analysis

Comparisons between two groups were conducted using Student’s *t* test, with statistical significance assessed at a significance level (α) of 0.05 (*P < 0.05, **P < 0.01, n.s.: not significant). The software programs of OriginPro 2019 and Microsoft Excel were used for statistical analysis and for creating the graphs, while R was additionally used for graph generation.
