# Supplementary material for: Autonomous chemo-metabolic construction of anisotropic cell-in-shell nanobiohybrids in enzyme-powered cell microrobots
Source: Sci Adv. 2025 Jun 25;11(26):eadu5451. doi: 10.1126/sciadv.adu5451 (PMC12189949; doi:10.1126/sciadv.adu5451)
Supplement: Supplementary file 1 — Figs. S1 to S10 [file sciadv.adu5451_sm.pdf]

Science Advances
AAAS

Supplementary Materials for

Autonomous chemo-metabolic construction of anisotropic cell-in-shell nanobiohybrids in enzyme-powered cell microrobots

Nayoung Kim *et al.*

Corresponding author: Insung S. Choi, ischoi@kaist.ac.kr

Sci. Adv. **11**, eadu5451 (2025)
DOI: 10.1126/sciadv.adu5451

This PDF file includes:

Figs. S1 to S10

Supplementary Figures:

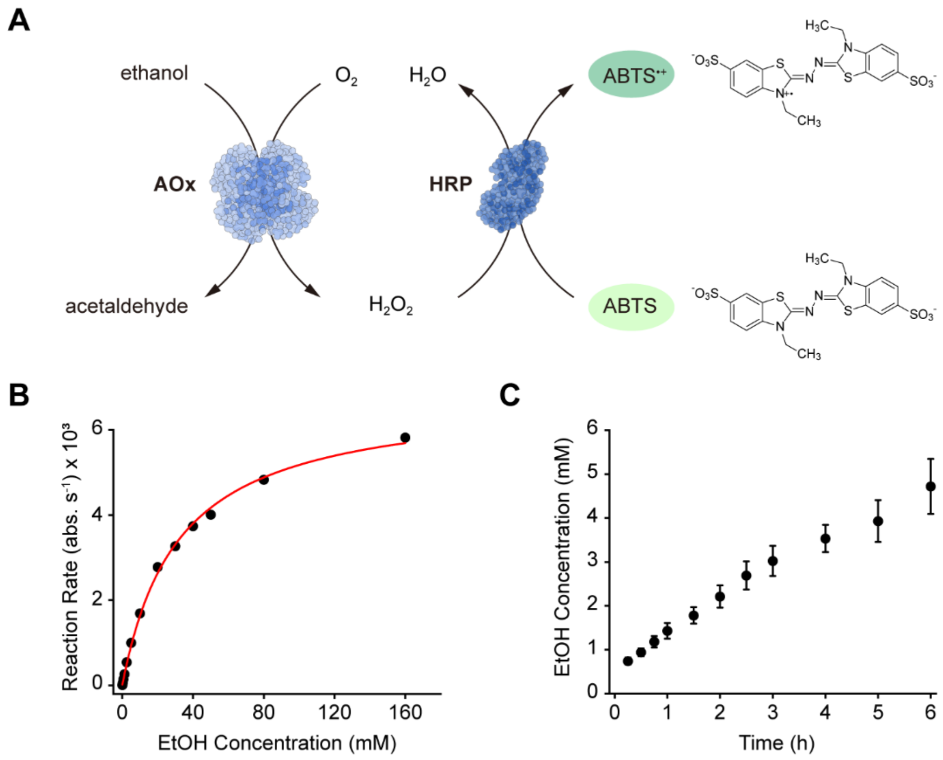

Fig. S1| Quantification of EtOH produced during yeast fermentation. (**A**) A schematic for EtOH quantification using the AOx-HRP cascade system. (**B**) Plot of the initial reaction rate with different EtOH concentrations. (**C**) Time course of EtOH production during yeast fermentation. The data are plotted as mean values ± SE (n = 3, from independent experiments).

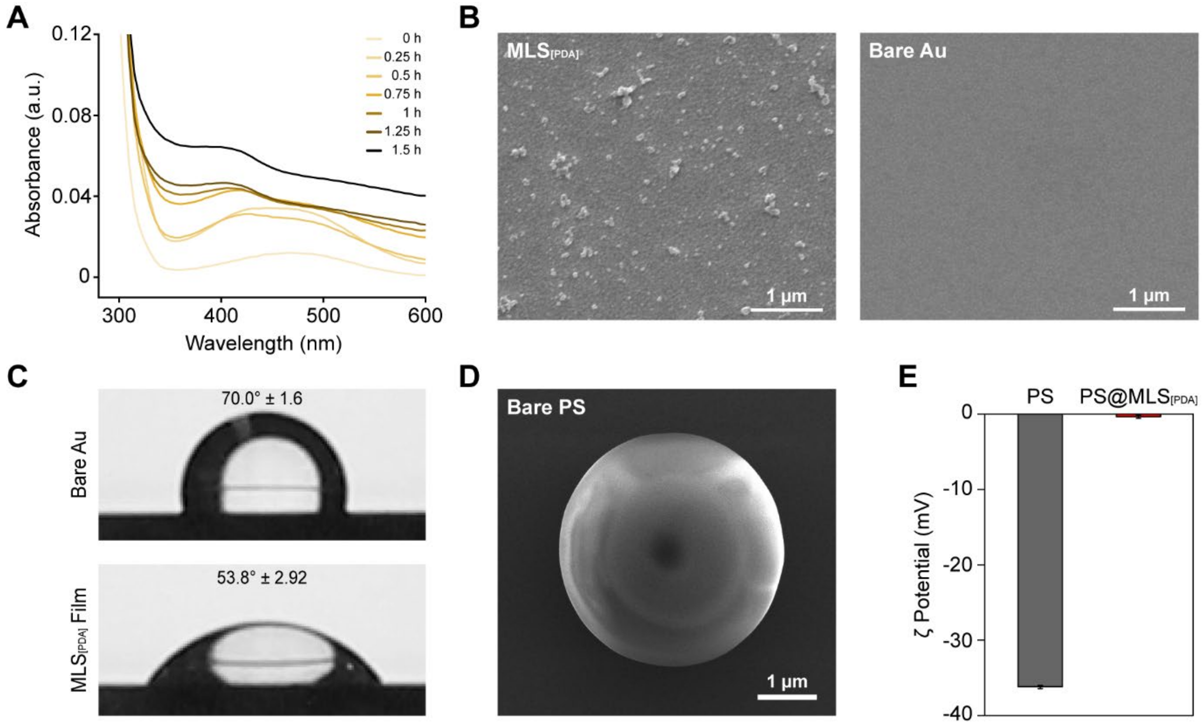

Fig. S2| Characterizations of MLS$_{[PDA]}$, MLS$_{[PDA]}$ films, and MLS$_{[PDA]}$ shells synthesized via AOx-HRP system. (**A**) UV-vis absorption spectra of a dopamine solution over reaction time with the AOx-HRP system. (**B**) FE-SEM images of the MLS$_{[PDA]}$ film on a gold substrate and a bare gold substrate. (**C**) Water contact angles, before and after MLS$_{[PDA]}$ film formation. (**D**) FE-SEM image of a bare PS microparticle. (**E**) ζ-potential changes of PS microparticles before and after MLS$_{[PDA]}$ shell formation. The data are presented as mean values ± SD (*n* = 3, from independent experiments).

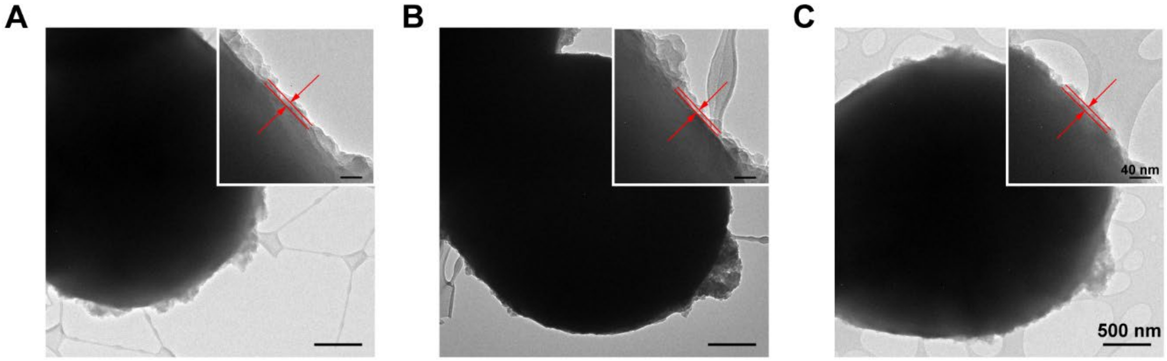

Fig. S3| TEM images of *S.cerevisiae*@MLS$_{[PDA]}$ constructed under different reaction conditions. (**A**) Control condition; (**B**) doubled concentration of D-glucose; (**C**) doubled concentrations of AOx and HRP.

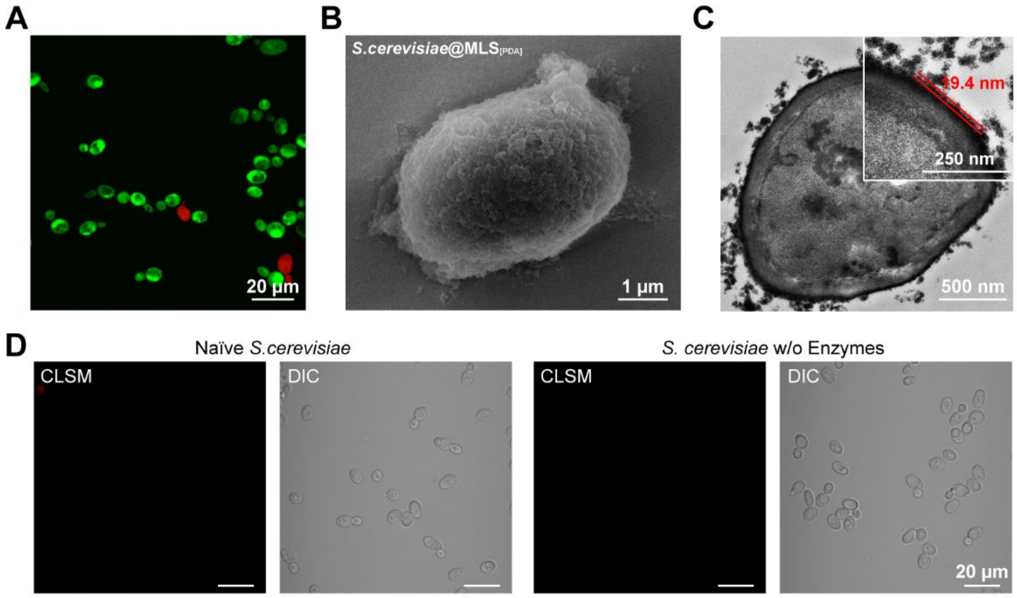

Fig. S4| Characterizations of *S. cerevisiae* after the reaction with or without the AOx-HRP system. (**A**) CLSM image of *S.cerevisiae*@$MLS_{[PDA]}$ synthesized with the pre-incubation of *S. cerevisiae* in a D-glucose solution. Green: live, red: dead. (**B**) FE-SEM image of *S.cerevisiae*@$MLS_{[PDA]}$, autonomously constructed without pre-incubation. (**C**) TEM micrographs of ultramicrotome-sliced *S.cerevisiae*@$MLS_{[PDA]}$ autonomously constructed without pre-incubation. (**D**) CLSM and DIC images of naïve *S. cerevisiae* and *S. cerevisiae* incubated in a dopamine solution without the AOx-HRP system: both incubated in the solution of BSA-647 prior to imaging.

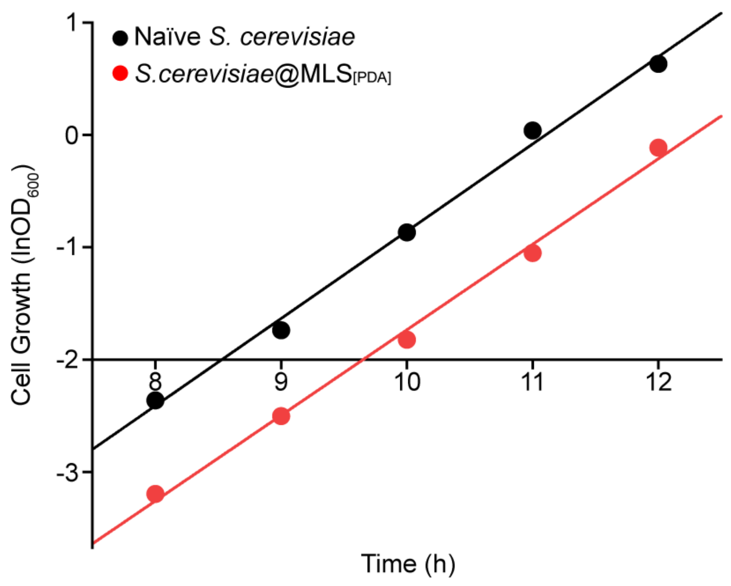

Fig. S5| Linear-fitted plots of ln(OD_{600}) of (black) naïve *S. cerevisiae* and (red) *S.cerevisiae*@MLS$_{[PDA]}$.

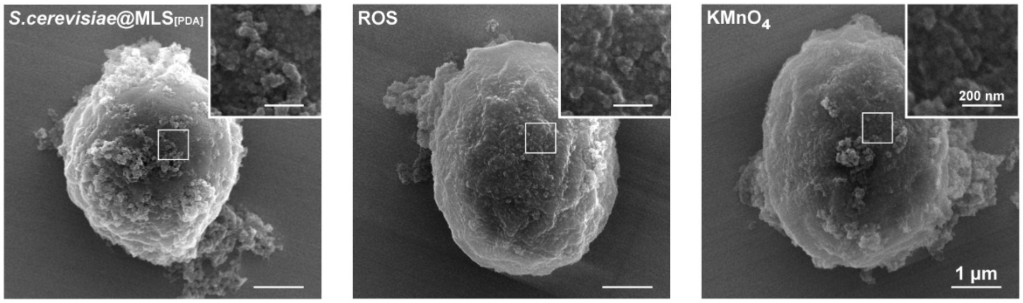

Fig. S6| FE-SEM images of *S.cerevisiae*@MLS$_{[PDA]}$ and *S.cerevisiae*@MLS$_{[PDA]}$ after the exposure to ROS and $KMnO_4$.

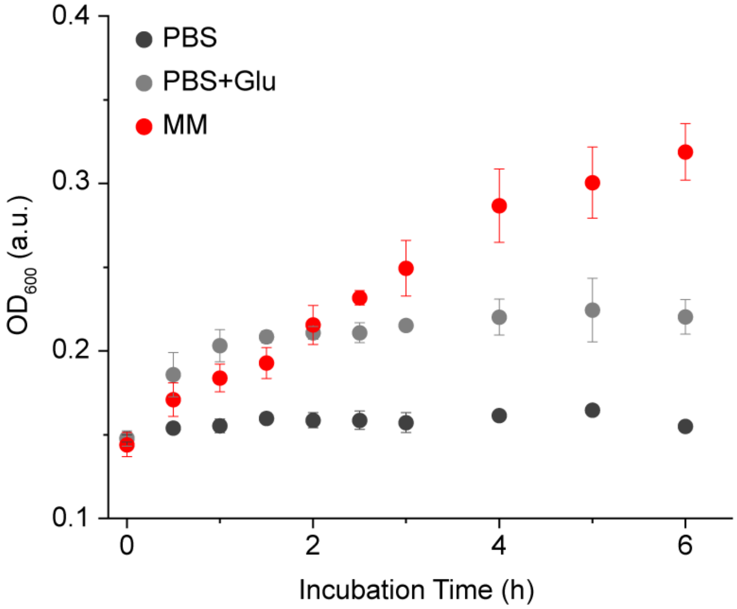

Fig. S7| Growth profiles of *S. cerevisiae* in various culture media. Graphs of the optical density at 600 nm (OD_{600}) over incubation time in three different culture media: PBS, PBS+Glu, and MM. The data are presented as mean values ± SD (n = 3, from independent experiments).

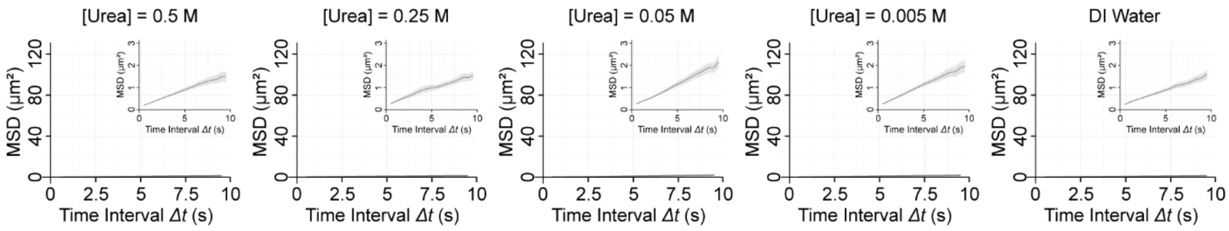

Fig. S8| Graphs of MSD versus time interval for naïve *S. cerevisiae* in urea solutions with different concentrations and DI water. The data are presented as mean values ± SE. The number of particles (*n*) is 68, 108, 83, and 73 for 0.5 M, 0.25 M, 0.05 M, and 0.005 M of urea, and 64 for DI water, respectively.

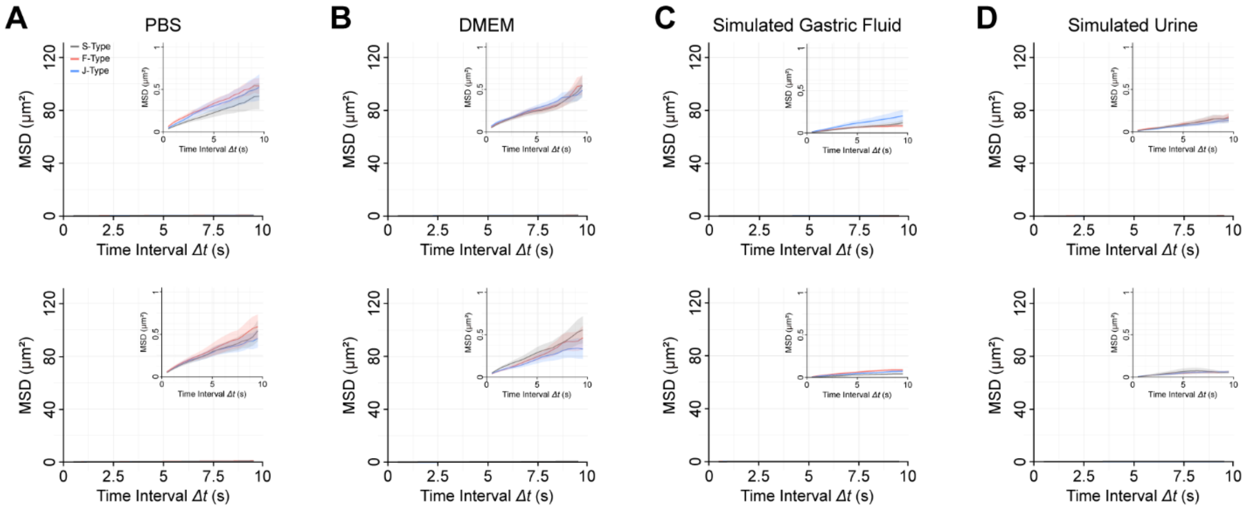

Fig. S9| Graphs of MSD versus time interval for S-, F-, and J-type microrobots in various simulated biological fluids at the urea concentration of 0.05 M (top) and without urea (bottom). The data are presented as mean values ± SE. In the case of PBS in the presence of urea, the number of particles (*n*) is 58, 45, and 46 for S-, F-, and J-types, respectively (**A**, top); for DMEM, 38, 38, and 51 (**B**, top); for simulated gastric fluid, 66, 46, and 49 (**C**, top); for simulated urine, 46, 40, and 41 (**D**, top). In the case of PBS in the absence of urea, the number of particles (*n*) is 48, 39, and 47 for S-, F-, and J-types, respectively (**A**, bottom); for DMEM, 30, 35, and 33 (**B**, bottom); for simulated gastric fluid, 60, 51, and 48 (**C**, bottom); for simulated urine, 33, 32, and 50 (**D**, bottom).

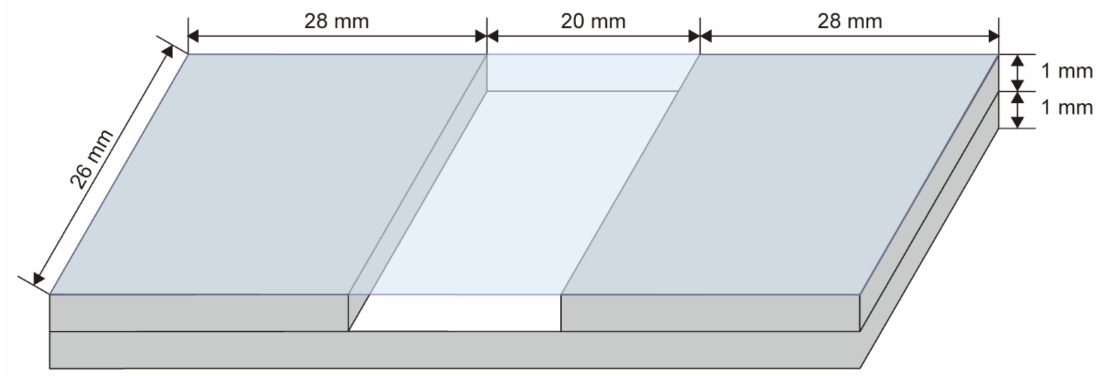

Fig. S10| Schematic of a homemade chamber used to observe the motional behaviors of cell microrobots. The chamber was constructed using slide glass and covered with a microscope cover glass. The reaction solution was introduced into the chamber.
